# Correlates of Mental Health Service Utilization in Married Women in Tehran 2011

**Published:** 2013

**Authors:** Masuod Ahmadzad-Asl, Farnoush Davoudi, Homa Mohammad Sadeghi, Noshin Khademolreza, Noushin Zarei, Morteza Naserbakht, Marziyeh Nojomi, Maryam Rasoulian

**Affiliations:** 1 Resident of psychiatry, Mental Health Research Center, Tehran psychiatry institute, Tehran University of Medical Sciences, Tehran, Iran.; 2 Resident of Community Medicine, Department of Community Medicine, School of Medicine, Tehran University of Medical Sciences, Tehran, Iran.; 3 Psychiatrist, Mental Health Research Center, Tehran psychiatry institute, Tehran University of Medical Sciences, Tehran, Iran.; 4 Specialist of Community Medicine, Mental Health Research Center, Tehran psychiatry institute, Tehran University of Medical Sciences, Tehran, Iran.; 5 Professor of Community Medicine. Department of Community Medicine, School of Medicine, Tehran University of Medical Sciences, Tehran, Iran.; 6 Associate professor of psychiatry. Mental Health Research Center, Tehran psychiatry institute, Tehran University of Medical Sciences, Tehran, Iran.

**Keywords:** Access to Services, Health Services Utilization, Mental Health, Perceived Need

## Abstract

**Objective:** There are disparities in mental health services (MHS) utilization within and between populations and several factors are studied as its potential correlates. Identifying those correlates would help health policy makers to adjust service provision with characteristics of their community. To evaluate demographic, socioeconomic and system correlates of MHS utilization among married women from Tehran, Iran.

**Methods:** A household survey of 615 married women residents of 22 municipal districts of Tehran selected via a cluster sampling method. All subjects were asked about health services utilization during last one and 12 months as well as need and access for MHS, demographic and socioeconomic factors. Independent correlates of MHS use were determined with logistic regression analysis.

**Results:** Total 615 women, mean±SE age and duration of marital life of 42.6±0.9 and 22±0.8 years, respectively were selected, rate of MHS utilization during last one and 12 months were 5.2% and 10.1% respectively. 23.6% of women reported having mental illness and 19.3% and 17.9% had need for MHS and access to outpatient health services, respectively. Logistic regression models showed that need for MHS (OR:5.25, 95%CI:2.7-10.1), access to outpatient services (OR:2.17, 95%CI:1.04-4.52), smoking (OR:3.4, 95%CI:1.16-10.2) and crowding index (OR:0.69, 95%CI:0.48-0.99).

**Conclusions: **Rate of MHS utilization in women are low considering the near to estimated rate of perceived illness. Bridging the gap between perceived illness and need for services, then providing better access to services in areas with higher crowding index and higher rates of smoking in residents should considered in any mental health promotion programs.

**Declaration of interest: **None.

## Introduction

Mental health services utilization could be an indicator of mental health status in community and met mental health needs. Addressing correlates of utilization helps reduce unmet need for mental health care (MHC), which might improve health and reduce costs for both physical and mental health care (1-3).

Perceived or documented history of mental and physical illness, as well as severity of current mental illness, are reported to be associated with higher rates of perceived need for care (4).

 An understanding of how citizens evaluate their mental health status and need for mental health services may help health care policy makers and managers evaluate and plan more effectively for MHC demographic ones and socioeconomic factors (5).

Increased access may not mean better health outcomes. Assessment of correlates of service utilization in health sector may be useful To ensure resource efficiency and avoid wastage, To assist the establishment of priorities in limited resource environments, To increase the possibilities for reducing health inequities and In general – to provide an appropriate evidence base for health planning (6-8).

Health demands are influenced by demographics; socio-economic status; health services supply; environmental situation – e.g. water, sanitation; nutrition; illness behaviors; life style; perceptions of illness & health expectations and trade-offs (9-10).

Health services utilization conceptualized among proximate factors that lead to health outcomes such as mortality, morbidity and disability (11-12). Several reports delineate the role of demographic factors such as age, sex, education, occupation, and household income/wealth as well as perceived need to mental health services (2) in health services utilization. All of these factors in aforementioned levels are among determinants of health in community (13-15). 

There are reports of disparities in mental health utilization among different community members and study of factors leading to these disparities and planning to eliminate disparities in mental health utilization was noted as to priority in MHC system management (16). Identification of correlates of mental health services utilization would help health policy makers and managers to have better view of MHC indices and do more evidence based planning for MHC system (17-19). The aim of this study is to evaluate demographic and socioeconomic correlates of MHC utilization along with other correlates such as need for MHC and access to MHC among married women from Tehran, Iran.

## Materials and Methods

This manuscript shows findings from “study of service utilization, depression and anxiety with domestic violence among married women in Tehran, Iran” project as a part of two psychiatric specialty dissertations of M.A.A and N.Z that is also granted project no. 89-04-121-12426 from MHRC, TUMS.

*Design and setting*

This study is a cross sectional household survey among married women from all 22 districts of Tehran metropolitan. Tehran, the capital city of Iran has about 8.5 million populations in 2011 that contains about 11 percent of population of Iran. Tehran has 22 municipal districts each with different socio-cultural status. There are 370 neighborhoods in these 22 districts. 

*Sampling*

We used 42 neighborhoods as sampling clusters each with 15 samples and then based on proportional population in each district we assigned between 1-3 clusters to each districts and then based on random number table we selected sampling units. In each cluster with clockwise turn and systematic random sampling method 15 residential units were selected and then interview with married women (the youngest one if there were more) of the unit was done about study measurements. If there were no response to ring or refusal to participate the next nearest unit were replaced. Among 630 initial samples finally we enrolled 615 completed samples in the study.

*Data collection*

Study data were collected by direct interview with married women in selected residential units. Interview was done at morning, noon, and afternoon hours proportionally to be assured about participation of all women with different status of presence at home. If there were no response on first ring the interviewer would came back during next three hours and if no response again, the next consecutive residential unit would replace. In case of refusal to participate, two efforts would take to encourage participation by explaining the valence of research in women’s health promotion and exploring possible barrier of participation and try to overcome it by giving information and assurance. If refusal continues, the next residential unit would be replaced.

Five interviewers were selected that were graduate bachelors and master’s degree of psychology and sociology. They trained in a two-hour workshop about concept and method of project, sampling and data collection to assure consistency and reliability. Five percent of samples were re-evaluated by an independent supervisor to confirm validity of sampling and interview process.

*Main measurements*

Participants asked about Health service needs; i.e. need for outpatient medical and consultation services during last month and need for inpatient services during last 12 months and the frequency that those needs occur; Service utilization; i.e. frequency of visiting a general practitioner (GP), psychiatrist, consultant/psychologists and receiving non-prescribed medications from pharmacy during last month and last 12 months. Mental health services (MHS) were defined as utilizing either psychiatrists or consultant/psychologists services.

 Demographic and socioeconomic variables also were asked from participants: age, education, job, health insurance status, household income, crowding index (number of households per room), smoking and substance use in past year (in subject and her spouse) and perceived mental and/or physical illness status (in subject and her spouse).

*Statistics and data analysis*

The analysis was done by SPSS for Windows 16.0 (SPSS Inc, Chicago, ILL). After initial data entry, an independent operator qualified data entry. Descriptive analysis revealed mean, frequency and standard error (SE), the correlated of mental health services used were defined by logistic regression analysis including demographic and socioeconomic factors as independent variable in the logistic regression model. In all analyses p<0.05 was considered significant.

We graphed distribution of MHC utilization indices in Tehran area by Arc view 3.2 (ESRI Inc.) to show geographic distribution.

## Results

In a household survey, we enrolled 615 married women, residing in 22 municipal districts of Tehran with cluster sampling method. Mean (±SE) age of women and their spouses were 42.6±0.9 and 47.8±0.6 years old, respectively. Mean marital life duration in current relationship was 22±0.8 years. Age at marriage was 19.7±0.2 years and 96.1% of participants reported that they are living their first marriage (and first marriage of their spouse in 92.5%). Education level high school and lower, high school diploma and college and higher were reported in 35.2%, 42.3% and 22.4% respectively and the corresponding educational level in spouses were 36.5%, 35.8% and 27.7%, respectively. About job status 82.4% were house workers for their own, 10.3% employed, 2.5% unemployed and 3.1% retired. The rate of employment in spouses was 89.2%, while 8.0% were unemployed, and 2.0% retired.

Table 1 has summarized demographic and socioeconomic factors of subjects.

Crowding index was 1.87±0.09 and mean household members were 3.97±0.05 person.

Subjects claimed themselves having mental and physical illness in 23.4% and 23.6% respectively and overall perceived mental/physical illness was reported in 38.5% of subjects. While reported mental, physical and either illnesses in spouses were 12.8%, 20.3% and 27.8% respectively. Only 3.7% of subjects reported having disabled/chronic diseased child in home.

Smoking, alcohol and substance use during last 12 months was found in 3.4%, 0.2% and 0.7% of women, respectively. Smoking, alcohol use and substance use in spouses during last 12 months was reported in 25.5%, 0.8% and 4.2%, respectively.

*Health insurance and access factors*

Among study subjects, 82.4% had health insurance coverage (52.2% social security insurance, 24.6% health services insurance) and 33.7% had complementary health insurance coverage. Perceived need for medical outpatient services and mental health services and inpatient services reported in 42.1%, 19.3% and 14.5% of subjects, respectively. Access to outpatient services in different degrees reported in 17.9% of subjects. 

*Mental Health services utilization and its correlates*

Table 2 delineates services utilization during last month and last 12 months in study subjects.

Rate of MHS utilization during last one and 12 months were 5.2% and 10.1% respectively.

Women utilized MHS, were about 14.4±3.8 years older than others (mean age 56.2±13 vs. 41.8±0.5 years old, p<0.01) and age of spouseof women visiting consultants were significantly younger that others (44.3±2.2 vs.48.1±0.5 years old, p<0.05).

**Table1:** Demographic, socioeconomic and health correlates of services utilization during last 12 months in women, Tehran, Iran, 2011-12

	Total^§^	Selected health services				
		Mental health				
		Psychiatrist	Consultant	Any	GP	Any
Total, n (%)	615 (100)	37(6.0%)	43 (7.0%)	62 (10.1%)	465(73.8%)	555 (90.2%)
Age; years old, mean±SE	42.6±0.9	56.2±13^‡^	39.1±1.9	47.8±7.8^‡^	42.8±1.2	42.7±1.0
Education level,						
High school and lower	35.2%	06.0%	04.2%	08.3%	75.5%	88.9%
High school diploma	42.3%	05.4%	06.5%	09.2%	72.7%	89.2%
College and higher	22.4%	07.3%	12.4%^†^	14.6%	73.7%	94.9%
Job,						
House-worker	82.4%	06.1%	06.7%	10.1%	74.5%	81.7%
employed	10.3%	04.3%	11.4%^†^	11.4%	72.9%	91.4%
unemployed	02.5%	13.3%^†^	00%	13.3%	66.7%	93.3%
retired	03.1%	04.5%	04.5%	04.5%	68.2%	100%
Household’s monthly income,						
Less than 4*1,000,000 Rls	23.7%	05.5%	05.5%	08.9%	72.6%	86.3%
4-7*1,000,000 Rls	38.4%	07.6%	05.9%	10.2%	77.5%	92.4%
More than 7*1,000,000 Rls	24.9%	05.2%	09.8%	11.8%	67.3%	90.8%
Not declared	13.0%	03.8%	07.5%	08.8%	77.5%	90.0%
First marriage,	96.1%	05.9%	06.9%	10.2%	74.3%	90.5%
Marriage duration, years, mean±SE	22±0.8	23.2±2.2	17.8±2.1^*^	20.8±1.9	21.6±0.7	21.5±0.6
No. of rooms in house, mean±SE	2.44±0.04	02.4±0.15	02.4±0.04	02.4±0.1	02.4±0.04	02.4±0.04
Crowding index, mean±SE	1.87±0.09	01.8±0.2	01.6±0.1^*^	01.7±0.1	01.9±0.05	01.9±0.05
No. of children, mean±SE	3.3±0.08	03.3±0.3	02.9±0.3	03.3±0.1	03.3±0.1	02.35±0.07
Personal living place	58.2%	05.9%	06.4%	09.8%	73.2%	90.2%
Having motor vehicle	66.2%	05.7%	06.6%	09.6%	72.2%	89.7%
Physical illness,	23.4%	04.8%	06.2%	09.0%	79.3^†^	90.3%
Mental illness,	23.6%	15.3%^‡^	11.8%^†^	21.5%^‡^	82.6%^‡^	92.4%
Illness (mental/physical) ,	38.%	10.5%^‡^	09.3%	15.6%^‡^	78.5%^†^	90.7%
Smoking^£^,	03.4%	19.0%^†^	19.0%^†^	28.6%^‡^	71.4%	85.7%
Substance use ^£^,	00.7%	00%	00%	00%	100%	100%
Need for medical services	42.7%	7.3%	10.0%^†^	13.1%^†^	78.0%	91.9%
Need for mental health services	19.3%	14.3%^‡^	17.6%^‡^	23.5%^‡^	79.8%	89.1%
Access to outpatient services	17.9%	08.2%	09.1%	16.4%^†^	75.5%	90.9%
Spouse						
Age; years old, mean±SE	47.8±0.6	50.2±2.5	44.3±2.2^*^	46.9±1.8	47.6±0.6	47.8±0.6
Physical illness,	20.3%	08.8%	06.4%	10.4%	75.2%	92.8%
Mental illness,	12.8%	12.7%^†^	16.5%^‡^	17.7%^†^	77.2%	94.9%
Illness (mental/physical) ,	27.8%	09.4%^†^	09.9%	12.9%	76.0%	93.6%
Smoking^£^,	25.5%	08.9%	10.2%	14.6%^†^	72.6%	91.7%
Substance use ^£^,	04.2%	03.8%	07.7%	11.5%	73.1%	84.6%

† Values in this column denote value in each subgroup among all study subjects.

‡ **GP**. General practitioner.

§ “**Any**” in selected health services denotes all health services (more than mentioned list) utilization in the table during last 12 months.

*. Significant at 0.05 level, lower in test group;Significant at 0.05 level, higher in test group;

‖ Significant at 0.01 level, higher in test group;

¶ during last 12 months.

**Table 2:** Frequency of service utilization among married women in Tehran, Iran, 2011-12

	**Service Type**	**Mean±SE**	**No. of Visits**	**% of all health services**	**No. Visits per 1000 population (95%CI** ^†^ **)**
**Last month**	**General Practitioner**	0.65±0.05	397	24.6%	646 (627-664)
	**Psychiatrist**	0.05±0.01	32	02.0%	052 (48-56)
	**Consultant/psychologist/psychoanalyst**	0.04±0.01	27	01.7%	044 (41-47)
	**Pharmacy, non-prescribed medication use**	0.82±0.05	504	31.2%	820 (808-831)
	**Mental health services**	0.10±0.02	59	03.7%	096 (89-103)
**Last 12 months**	**General Practitioner**	2.7±0.1	1655	38.0%	2691 (2331-3051)
	**Psychiatrist**	0.19±0.04	114	02.6%	0185 (173-197)
	**Consultant/psychologist/psychoanalyst**	0.21±0.05	131	03.0%	0213 (200-226)
	**Mental health services**	0.40±0.07	245	05.6%	0395 (376-414)

† . CI: Confidence Interval

Spouse smoking and substance use was correlated to more pharmacy use by women (62.5% vs. 39.3% in women with substance use and substance null spouse Smoking women had more visits with psychiatrists (19% and 5.6% of smoking and non-smoking women, respectively) and consultants (19% and 6.6% of smoking and non-smoking women, respectively) during last 12 months (but not last months); P<0.05. Smoking women utilized more mental health services (28.6%) compared to non-smoking women (9.5%); p<0.01. and 48.1% and 37.6% in women with smoking and non-smoking spouse, and respective, p<0.05). Other services utilization did not differ based on smoking and substance use in women and their spouses. 

During last month subjects with mental illness utilized 14.6% more GP services (50.7% vs. 36.1% in last month, in subject with and without mental illness, p<0.01), 10.2% more psychiatrist services (11.1% vs. 0.9% in subject with and without mental illness, p<0.001), 3.5% more consultant services (5.6% vs. 2.1% in subject with and without mental illness, p<0.05) but not pharmacy services, (44.8% vs. 38.8% in subject with and without mental illness, p<0.01). MHS utilized in 14.6% of subjects that reported having mental illness. 

During last month also subjects with reported physical illness compared to subjects without physical illness also utilized more GP services (47.7% vs. 34.4%, respectively; P<0.001), Psychiatrist services (7.2% vs. 1.1%, respectively; p<0.001) but not consultant services (4.2% vs. 2.1%, respectively; p>0.1). Among subjects with physical illness reported more utilization of MHS during last month (9.7% vs. 2.4%, respectively; p<0.01). 

Services utilization during last 12 months also showed significant differences between subjects with and without mental/physical illness (Table 1) subject with mental illness utilized 11.6% more GP services (82.6% vs. 71.0%, respectively; p<0.01), 12.3% more psychiatry services (15.3% vs. 3.0%, respectively; p<0.001), 6.2% more consultant services (11.8% vs. 5.6%, respectively; p<0.05) and 15.1% more MHS (21.5% vs. 6.4%, respectively; p<0.001). 

Considering service utilization in last 12 months, having physical illness showed no significant correlation with different group of services utilization (in all cases p>0.05). Subjects with physical illness utilized 8.1% more GP services (p>0.05), 1.4% less psychiatry services (p>0.05), 1.0% less consultant services (p>0.05) and 1.6% more all services (p>0.05).

Subjects with college and higher education level reported more consultant service use (12.4%,p<0.05) but not psychiatry or other groups of service use. Employment status, marital life duration, being in first marriage, showed no correlation with service use in different groups. Household’s income level and crowding index showed no significant correlation with different group of services utilization. 

*Independent correlates of mental health services utilization*

Logistic Regression models revealed that perceived need for mental health services (Odds ratio; OR: 5.25), perceived having mental illness (OR: 2.29), smoking (OR: 3.44), access to outpatient health services (OR: 2.17) and crowding index (OR: 0.69) are independent determinants of mental health services utilization in married women in Tehran. Age, age of spouse, physical illness, education level, job status, household’s income, spouse’s mental/physical illness and moking/substance use showed no Independent correlation with mental health services utilization(Table 3).

**Figure 1:**
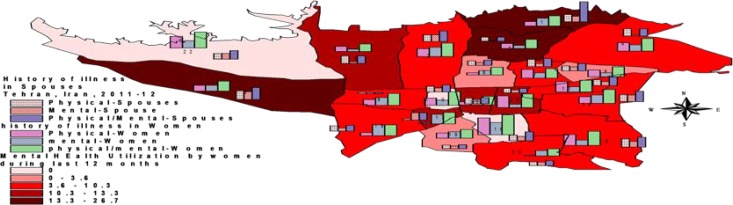
Distribution of mental health services utilization and perceived mental/physical illness in women and their spouses in Tehran municipal district, 2011-12

**Figure 2:**
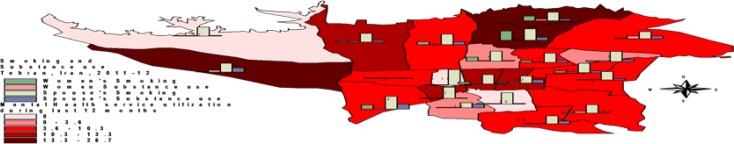
Distribution of mental health services utilization and smoking/substance use during last months in women and their spouses in Tehran municipal district, 2011-12

Geographic distribution of mental health services use in Tehran municipal districts and distribution of perceived having illness and smoking/substance use status in Tehran have plotted in figures  1 and 2.

**Table 3:** Logistic regression model of correlates of mental health services use among married women in Tehran, 2011-12

	Total	OR^†^ (95% CI^‡^)	P. value
Need for MH* services, Perceived	19.3%	5.25 (2.72-10.11)	<0.001
Age	42.6±0.9	1.01 (0.99-1.03)	00.190
Mental illness, Perceived	23.6%	2.29 (1.16-4.53)	00.017
Smoking	03.4%	3.44 (1.16-10.20)	00.026
Spouse’s Age	47.8±0.6	0.98 (0.93-1.03)	00.375
Crowding index^**^	01.87±0.09	0.69 (0.48-0.99)	00.043
Spouse’s Smoking	25.5%	1.46 (0.67-3.19)	00.340
Access to outpatient services, Perceived	17.9%	2.17 (1.04-4.52)	00.040
Constant	-	0.154	00.002

† . Odds Ratio;

‡ . CI: Confidence Interval;

§ . Mental Health;

‖ . Crowing index: ratio of household’s members to rooms.

## Discussion

Our study showed several determinants of mental health utilization and disparity and frequency of MHS utilization in married women in Tehran. Study of health service utilization could help policy makers, managers and directors in health sector. These data are useful in determining outreach populations, correlates of services use, over/under use of services based on global standards and unmet needs, and estimation of resources need for service provision (20-21). 

Service utilization depends on several factors: demographic factors (age, marital status, family width, education level, employment, and socioeconomic status), insurance and social support systems, access to services, acceptability of services, and need for serviced (perceived/real) (22-23). 

Our findings showed relatively low rate of mental health services utilization among women in Tehran that would reflect also an optimistic picture of MHS utilization in Iran as well. Only 10.1% of women have utilized psychiatric and/or psychological services in last year, considering perceived mental illness in 23.4% and need for MHS in 19.3%, it is clear that there is large gap of unmet need for services in MH system. Rate of MHS use have reported in wide range between 6.5% (in elderly) and 34.5% (in college students) during last year (20, 24-25). Subjects with mental illnesses (26-27), living in stressful conditions such as immigrants (28-29)and victims of crimes such as rape or violence reported higher rates (between 52%-72.5% in victims of rape (30-31), 37% in victims of intimate partner violence (32-33). Need for MHS in our study were similar to study in California in 2005 (34).

About 76.5% of women who declared need for MHS did not used any MH facility in last year, on the other hand need for MHS was the most potent predictor of MHS use in women (OR: 5.25). This finding emphasizes both planning to need management programs and bridging gap of unmet need in MH system promotion. Note that only 32.6% of women who had mental illness had perceived need of MHS (15.2% in women without mental illness, p<0.001) and the gap between perceived illness and need for services should be considered as a point of intervention in MH system promotion. 

Need for MHS in women estimated to be more than 1.5 timed than men (34), so it could estimated about 13% need for MHS in men. Noorbala et al. reported the rate of mental illness in women and men in Tehran in 2009, 37.9% and 28.6%, respectively (35). So we may have about 15-18% gap between illness and need for service in Tehran and considering that about 67.4% of women with perceived illness did not had any perceived need for MHS, this gap between health condition and need for services would be even more. So it seems that health system should plan to overcome this gap. Need for service is one of primary steps in the way of reaching fulfilled needs and mental health promotion programs. Comparing to Noorbala etal.(35). that used the same geographic area for sampling with our study and similar sampling method, the perceived having mental illness (23.4% of women) and real mental health condition (37.9% of women with mental illness) also shows a significant wide gap that need attention by mental health authorities [36]. This may highlights by the reports that the trend of mental illness seems to have incremental pattern in Tehran during last decade (37).

The rate of need fulfillment and service use in MH care varies in different countries and among different populations [9, 38-39]. In our study, the disparity of MHS utilization in Tehran geographic areas varied between about zero to 26% (average 10.1%) and this reflects inequities in MHS provision and marketing programs that have been pointed as first priority of MH system to overcome (3, 16, 25-26, 38, 40).

In this study, we found perceived need for MHS, access to outpatient health services, smoking, perceived mental illness, and a socioeconomic determinant i.e. crowding index to be correlated with mental health services utilization and these items could be used in planning mental health promotion programs in Iran. Socioeconomic factors have already been reported as one of major correlates of MHS use in literature (20, 31, 41-44).

Providing better access to services in areas with higher crowding index and higher rates of smoking in residents as well as need management programs to overcome gaps between perceived illness and need for services as well as unmet need for MHS should considered in any mental health promotion programs.

*Limitations*

In this study, we did not assessed some predisposing factors (e.g. attitude to MH), and need factors (e.g. mental health condition, severity of illness) on service utilization. Method of study that gathered data from community while conforming several advantages (such as better sampling coverage, community based design, and more real view of health condition of community) simultaneously suffers from some disadvantages such as sampling biases and issues on accuracy of reports. 

## Authors’Contribution

MAA, MR and NZ conceived and designed the evaluation and helped to draft the manuscript. MAA, MR, FD and HM participated in executing the evaluation, collected the clinical data, interpreted them and performed the statistical analysis. FD, HM, NK, MNb and MN re-evaluated the clinical data and revised the manuscript. All authors read and approved the final manuscript.

This paper is based on results from two dissertations for psychiatry specialty degree of MAA and NZ and grant no. 89-04-121-12426of MR from MHRC, TUMS.
